# Memory Effect by Melt Crystallization Observed in Polymorphs of a Benzothieno-Benzothiophene Derivative

**DOI:** 10.1021/acs.cgd.3c00847

**Published:** 2023-10-16

**Authors:** Ann Maria James, Alessandro Greco, Félix Devaux, Nemo McIntosh, Patrick Brocorens, Jérôme Cornil, Priya Pandey, Birgit Kunert, Lucia Maini, Yves Henri Geerts, Roland Resel

**Affiliations:** † Institute of Solid State Physics, Graz University of Technology, Petersgasse 16, 8010 Graz, Austria; ‡ Max Planck Institute for Polymer Research, 55128 Mainz, Germany; § Laboratoire de Chimie des Polymères, Université Libre de Bruxelles (ULB), 1050 Bruxelles, Belgium; ∥ Laboratory for Chemistry of Novel Materials, University of Mons, 7000 Mons, Belgium; ⊥ Dipartimento di Chimica “G. Ciamician”, University Bologna, 40126 Bologna, Italy; # International Solvay Institutes of Physics and Chemistry, Université Libre de Bruxelles, 1050 Bruxelles, Belgium

## Abstract

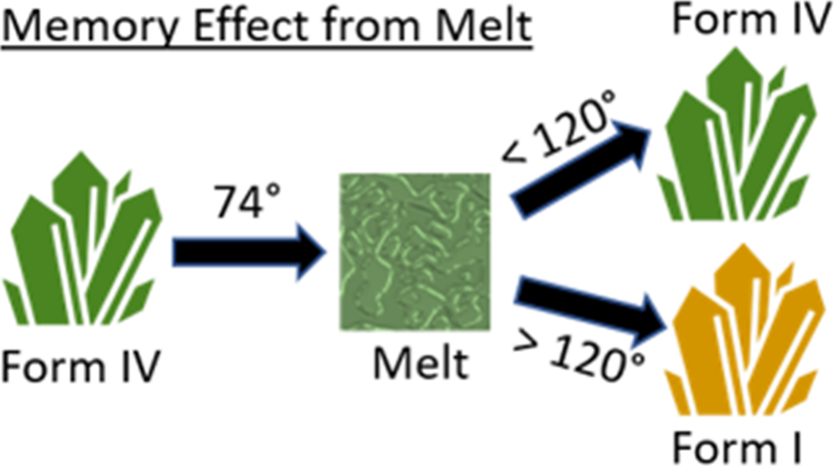

This work provides a comprehensive illustration of a crystalline melt memory effect recorded for three solvates of the 2,7-bis(2-(2-methoxyethoxy)ethoxy)benzo[*b*]benzo[4,5] thieno[2,3-*d*]thiophene (OEG-BTBT) molecule with dichloromethane (DCM) molecules. Combined optical microscopy and X-ray diffraction measurements at different temperatures are used to get an overview of the structural and morphological properties like melting points, isotropic transition temperatures, induction times, and crystallization kinetics of the three forms. An outstanding observation is made upon annealing the three polymorphs at temperatures well above their respective melting points as well as above the optical clearance temperature. After cooling back to room temperature, recrystallization results in the formation of the initial phase present before the annealing process. This melt memory effect is observed for all three solvates. These observations can be correlated to the strong interaction between the DCM molecules and the oligoethylene glycol side chains, even in the molten state. This conclusion rationalizes the experimental observation made upon solvent vapor annealing of the crystalline sample with DCM, which unambiguously transformed the system into a disordered state.

## Abstract 2

A melt memory effect was found for solvates of a molecular crystal with dichloromethane. Annealing at the isotropic state is accompanied by recrystallization in its initial phase as a solvate. Only sufficiently high temperatures result in a transfer into a nonsolvated crystal structure. The strong interaction of the solvent with oligo-ethylene-glycol chains might be responsible for the melt memory.

## Introduction

Being the active components for various electronic device applications demanding charge transport, light emission, and energy storage, extended π-conjugated molecules are key facets of organic semiconductors.^1,2^ The packing of these molecules in the solid state results in an overlap of the molecular orbitals, enabling charge transport.^3−5^ Due to their rigid molecular backbone, unsubstituted π-conjugated molecules are weakly soluble in common solvents, which impedes the easy preparation of devices by solution processing. However, the combination with flexible chemical groups (e.g., by attaching flexible side chains to the conjugated molecule) provides sufficient solubility while maintaining the semiconducting properties.^6,7^ Frequently used side chains for π-conjugated small molecules are alkyl or alkoxy groups.^8,9^ However, the introduction of side chains to a given molecular entity can trigger changes in their molecular packing.^10^

**Figure 1:**
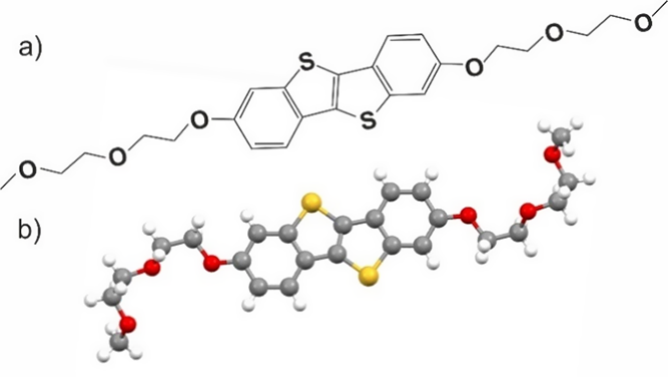
(a) Chemical structure of OEG-BTBT and (b) conformation of the molecule within the thermodynamically stable phase (Form I) at room temperature.

This work focuses on a molecule with a benzothieno-benzothiophene (BTBT) conjugated core and two oligoethylene-glycol (OEG) side chains attached at the terminal ends of the BTBT unit.^11^ The chemical structure of the molecule is depicted in Figure 1a. The choice of OEG as side chains aims to make the molecule susceptible to polar solvents. The crystallization of OEG-BTBT has been already studied by bulk polymorph screening as well as by crystallization within thin films.^12,13^ In the bulk, two enantiotropically related phases are identified by reversible temperature-dependent experiments; Form I represents the stable phase at temperatures below 403 K and Form II is stable from 403 to 429 K. The classical screening technique also identified a metastable solvate phase (Form III) from dichloromethane (DCM). Furthermore, crystallization at surfaces unveiled four additional phases (solvates with DCM or 1,2-dichlorobenzene),^13^ and the corresponding melting temperatures of the forms discussed in this paper are given in Table 1.

**Table 1:** Properties of the Five Different Phases of the Molecule OEG-BTBT: the Melting Temperature of the Crystalline Phases (Observed by XRD), the Temperature for the Isotropic Transition (Observed by Hot-Stage Optical Microscopy), the Number of DCM Molecules per OEG-BTBT Molecule,^13^ and the Order of Appearance by Crystallization from the Amorphous State

	Form I	Form II	Form IV	Form V	Form VI
melting temperature [K]	403	429	347	348	366
isotropic transition temperature [K]		433	376	404	413
DCM/OEG-BTBT	0	0	3	2	1
appearance from the amorphous state			1st	2nd	3rd

The molecular packing of Form I is dominated by the intermolecular interactions between the BTBT units of the molecules, promoting a herringbone packing of the aromatic units stabilized by quadrupolar electrostatic interactions.^14^ A wide variety of possible conformations of the OEG side chains have been reported in the literature. Helical conformations are predicted and witnessed in single crystals for long OEG chains,^15,16^ whereas all-trans conformations and irregular arrangements have also been reported.^17,18^ The side chain conformation within the crystal structure of OEG-BTBT (Form I) is depicted in Figure 1b. Gauche conformations are found for the dihedral angles OC–CO; such arrangements of OEG chains are identified as the lowest in energy for molecules in solution.^19^

This work demonstrates a crystalline melt memory effect, that is, the ability of a given polymorph to recrystallize from the melt into its pristine form. This ability suggests that even after heating well above the melting point, temperature-induced nucleation into the initial crystal structure is observed upon cooling below the melting temperature.^20^ This intriguing phenomenon is frequently observed during the crystallization of slowly crystallizing materials like polymers and glasses.^21,22^ In the case of polymer crystallization, a specific polymorph could appear at temperatures far above the melting point due to heterogeneous nucleation at impurities, by homogeneous nucleation from the internal structure in the melt (self-nucleation), or slightly above the melting temperature due to crystal fragments in the melt (self-seeding).^23−25^ However, the melt memory effect is hardly known for molecular crystals, despite the fact that it can be of technological importance.^24,26−28^ Here, the melt memory effect is evidenced for three different solvates of the molecule OEG-BTBT; recrystallization back to their initial structure is observed even after annealing far above their melting temperatures.

## Experimental Section

Thin films of the molecule 2,7-bis(2-(2-methoxyethoxy)ethoxy)benzo[*b*]-benzo[4,5]thieno [2,3-*d*]thiophene (OEG-BTBT) were prepared by solution processing. The as-synthesized material was dissolved in DCM at a concentration of 1 g/L or at a higher concentration of 4 g/L. The 250 μL of as-prepared solutions were drop cast on 2 cm × 2 cm silicon wafers with 150 nm thick thermally grown oxide coating. Similarly, the samples for hot-stage microscopy were prepared on optically transparent microscopy slides. Prior to the deposition, the substrates were chemically cleaned by ultrasonication in acetone for 15 min, followed by rinsing with isopropanol and drying with CO_2_ gas.

In order to get crystalline phases of the three considered solvates (Forms IV, V, and VI) of the molecule OEG-BTBT, the drop-casting procedure was varied by changing the concentration, reducing the evaporation rate of the solvent, or increasing the temperature of the substrate. Detailed sample preparation parameters are given elsewhere.^13^ Solvent vapor annealing was performed by keeping phase-pure thin films in the vapor of DCM for a period of 2 days.

The fabricated thin films were initially characterized by X-ray diffraction (XRD). The experiments were performed under specular scattering geometry, which at low scattering angles (2θ) reveals Bragg peaks from crystallographic planes that are oriented parallel to the surface of the substrates. The measurements were performed on a PANalytical Empyrean diffractometer. A sealed copper tube was used in combination with a multilayer mirror for monochromatization (λ = 1.5418 Å) and to produce a parallel beam. The diffracted beam was detected at the secondary side via a slit system (0.1 mm antiscatter slit/0.02 rad Soller slit) and a PANalytical PIXcel detector operating as a point detector. The angular measurements (2θ) were converted to the reciprocal space using the expression *q*_z_ = 4π/λ sin θ, where *q*_z_ is the scattering vector perpendicular to the substrate and 2θ is the scattering angle. The observation of Bragg’s peaks is analyzed in terms of peak positions, from which the corresponding interplanar distance (*d*) of a net plane series can be calculated as *d* = 2π/*q*_z_. In situ crystallization studies were performed by using a DHS900 heating stage under inert (nitrogen) conditions.^29^ The films were annealed for 30 min at a defined temperature (in between 373 and 413 K) to reach equilibrium^30^ and subsequently cooled down back to room temperature, with defined heating/cooling rates of 4 K/min. The recrystallization was monitored on the basis of XRD measurements, with a single scan taking 3 min.

The thin film morphology was investigated by optical microscopy (Zeiss, Model Axioskop 2 MAT) to examine the morphologies of different thin film solvates. The thin film samples were also investigated as a function of temperature by hot-stage microscopy. The thin film samples deposited on glass substrates were positioned in a heating chamber on an Olympus BX41 stereomicroscope equipped with a Linkam LTS350 platinum plate (for viable temperature control) and a VisiCam analyzer. The heating chamber was covered with a sealable cap during the heating and cooling cycles, and the rate was kept constant at 10 K/min. For all in situ experiments, time-lapse images were collected using a Nikon DS FI3 high-speed camera, and the recorded images were analyzed using the software Nikon NIS-Elements and Linksys32 data capture. The estimated error in the temperature measurement is lower than 10 K.

## Results

**Figure 2:**
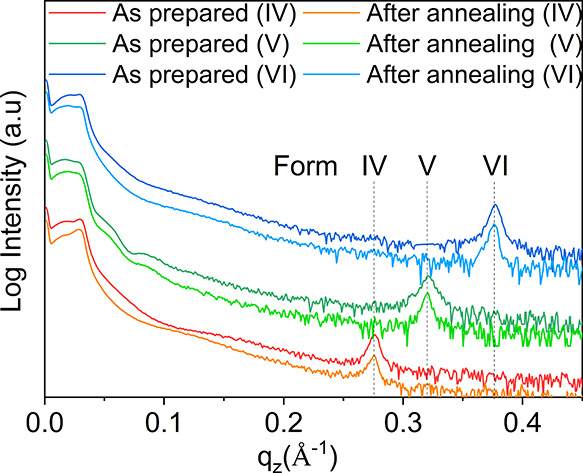
XRD pattern of OEG-BTBT of Form IV, Form V, and Form VI in the as-prepared state and in the recrystallized state after heating into the isotropic state. The films are prepared by drop casting on glass substrates. The curves are vertically shifted for visibility.

**Figure 3:**
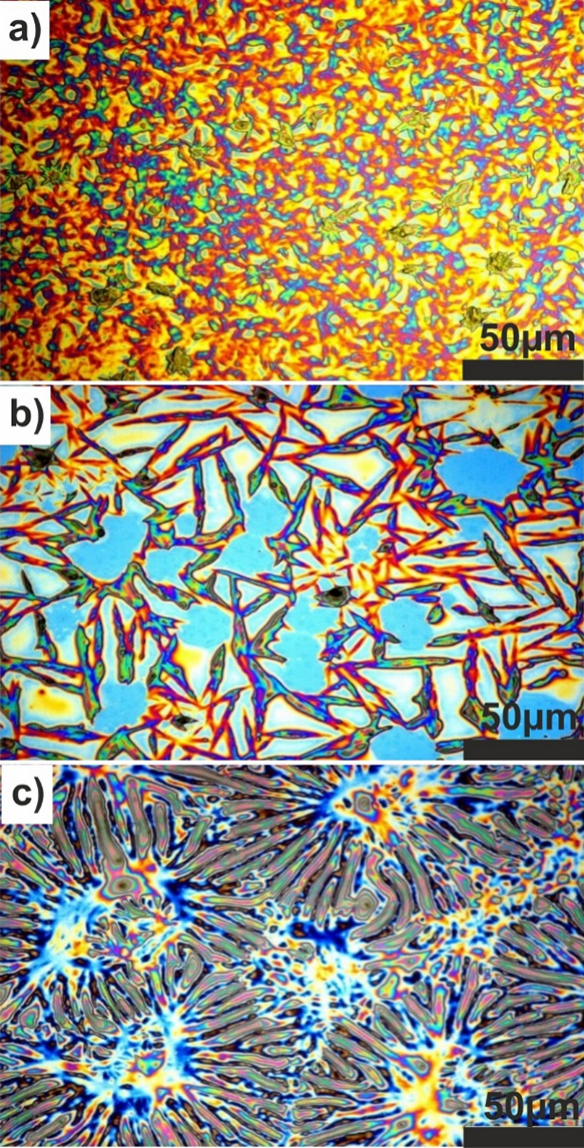
Optical microscopy images recorded for the three different solvates (a) Form IV, (b) Form V, and (c) Form VI of OEG-BTBT thin films on silicon substrates.

Surface crystallization is a potential approach to discover new polymorphs of a compound, which is exclusively found in the vicinity of a substrate surface. There are two reasons, first thin film preparation offers a variation over a broad range of the crystallization conditions, resulting in the appearance of exclusive polymorphs, and second, crystal nucleation at surfaces can be associated with confinement of the molecular packing with the substrate surface.^31−33^ As the first step of our investigations, thin films were prepared using defined growth parameters.^13^ The XRD patterns of the phase-pure thin films of Forms IV, V, and VI are presented in Figure 2 (glass substrates) and in Figure S1 (silicon substrates). No clear difference is observed between the two types of substrates. The morphology investigation performed by optical microscopy reveals unique characteristic features for all three forms. As seen in Figure 3, Form IV films show a rather homogeneous appearance, Form V films have random needlelike structures with a characteristic length of 50 μm, and Form VI films show ridgelike morphologies aligned toward central points.

**Figure 4:**
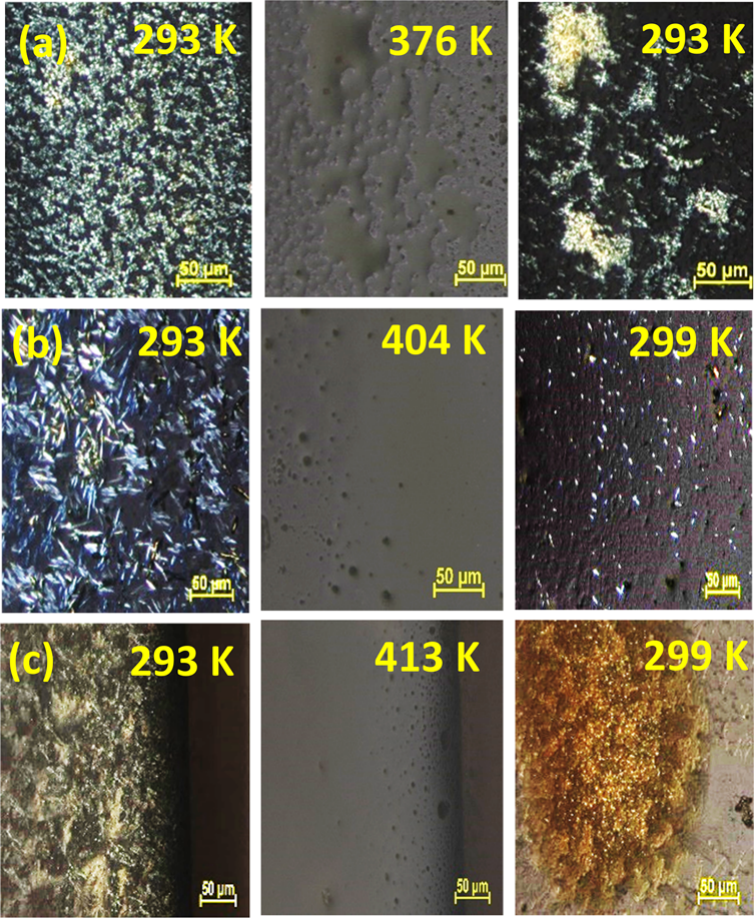
Hot-stage optical microscopy images recorded on solvates observed in thin films of (a) Form IV, (b) Form V, and (c) Form VI in the as-prepared state at room temperature (left column), at the clearing temperature while transitioning into an isotropic state (middle column), and after cooling back to room temperature (right column).

An extensive investigation of the three solvates of OEG-BTBT was further carried out by hot-stage microscopy using crossed polarizers. The change in morphology was followed as a function of temperature until the full extinction of the transmitted light. Figure 4 shows the starting morphologies of the thin films (left column), which are identical to those on silicon substrates (Figure 3). The morphology at a temperature within the isotropic state is given in the middle column of Figure 4. The observed transition temperatures are considerably larger than the melting temperatures observed by XRD^13^ (compare Table 1). Therefore, the observed transition might be correlated to a clearing temperature that appears after the breakdown of the remaining orientation order for neighboring molecules (e.g., as in a liquid crystalline state). After reaching the isotropic transition, the films were cooled back to room temperature. Recrystallization of the Form IV sample was observed at a temperature of 313 K, while the other two films (Form V and Form VI) showed delayed recrystallization at room temperature in the range of several hours.

Unforeseen and astonishing results were obtained after analyzing the XRD spectra recorded for the recrystallized films since the three solvates returned back to their respective initial phases rather than to the thermodynamically stable phase (Form I). Figure 2 shows the XRD patterns recorded before and after the heat treatment for the different polymorphic thin films. For other systems, a similar memory effect was already observed for crystallization from melt, although it is also known that the heat treatment at higher temperatures or elongation of the annealing time cancels this effect.^24^

A series of experiments were performed on phase-pure Form IV samples after annealing above the melting temperature and then cooling them to room temperature. The annealing temperature was varied between 373 and 393 K for a period of 30 min for equilibration.^30^ An additional series of measurements were performed at a temperature of 383 K by variation of the different annealing times between 30 min and 2 h. In all cases, the memory effect was observed (Figure S2c).

**Figure 5:**
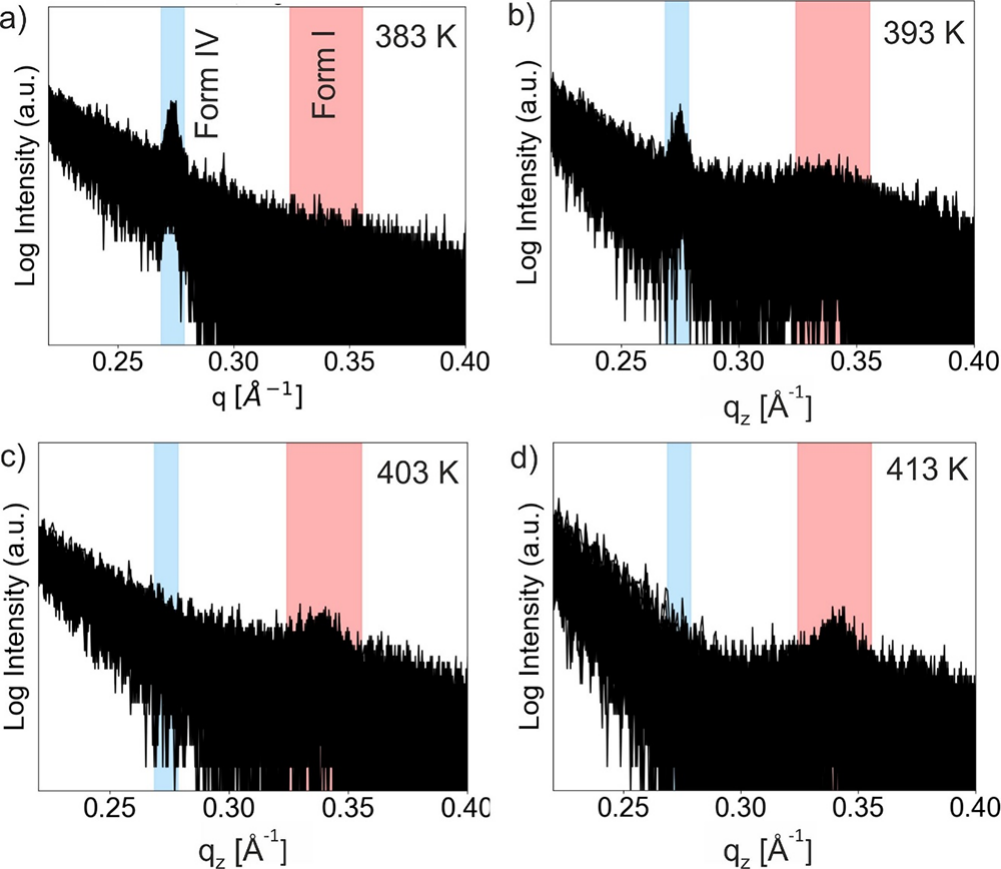
Temperature-dependent in situ XRD measurements of the recrystallization process of a Form IV film starting from different annealing temperatures of (a) 383, (b) 393, (c) 403, and (d) 413 K. A superposition of a large number of scans is depicted.

**Figure 6:**
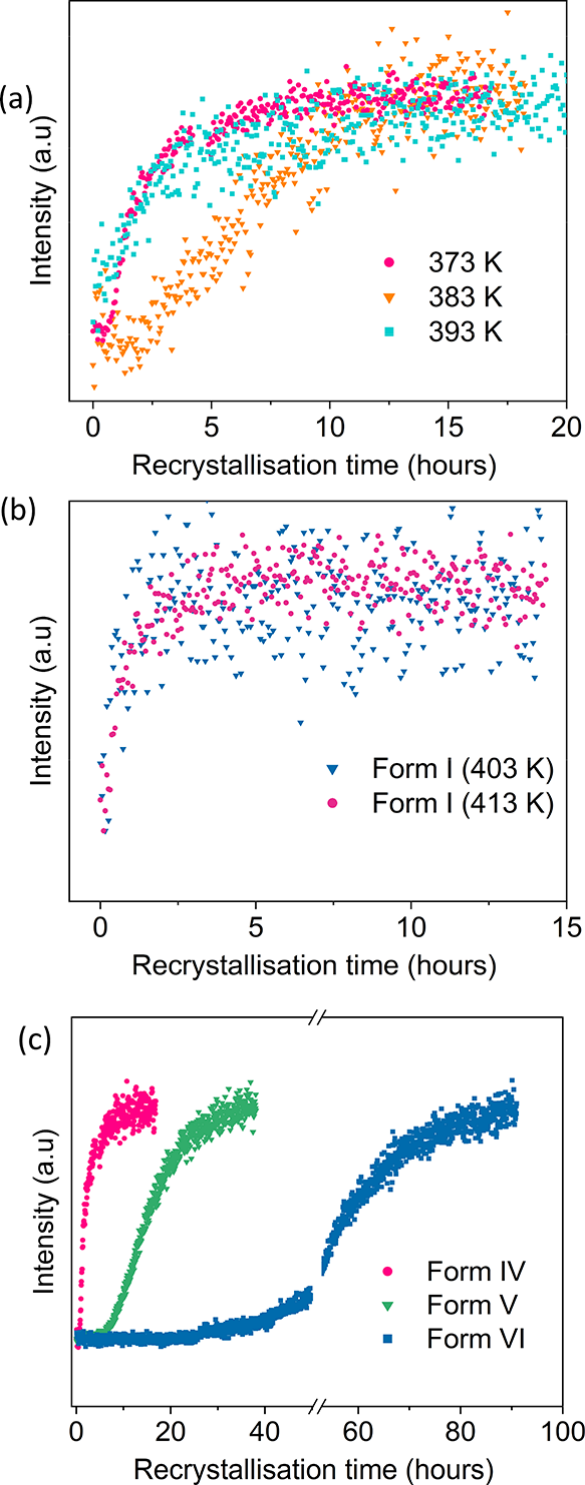
(a) Intensity of the 001 Bragg peak of Form IV during the recrystallization process after annealing at 373 K (magenta), 383 K (orange), and 393 K (blue). (b) Intensity of the 100 Bragg peak of Form I after heating a Form IV film to 403 and 413 K. (c) Characteristic recrystallization curves after annealing the films of the three different solvates (or polymorphs) at a temperature higher than their respective melting point (Form IV at 373 K, Form V at 363 K, and Form VI at 403 K). The curves are normalized to the maximum of the finally reached peak intensity.

Figure 5 shows in situ measurements during recrystallization. The Form IV thin films were annealed at temperatures between 373 and 413 K and subsequently cooled down to room temperature. The intensity of the 001 Bragg peak was monitored. In Figure 6a, the peak intensities of the Form IV recrystallization process are plotted as a function of time for annealing temperatures of 383 and 393 K; these temperatures are chosen above the melting temperature (347 K) and close to the isotropic transition (376 K). The curves reveal a characteristic time for the onset of crystallization (induction time) as well as the rate of crystal growth. The induction time is described by the time elapsed until the first observation of the Bragg peak while the slope of the curves above the onset of crystallization represents the crystallization rate. Although the samples show a variation of these two parameters, a clear relation to the annealing temperatures could not be found, because as evidently seen in the three chosen recrystallization curves, the intermediate annealing temperature yields the longest induction time. Nevertheless, it is quite well known that these two parameters can scatter in a vast range for defined crystallization parameters.^34−36^

The annealing experiments conducted on the Form IV thin film well above the isotropic transition temperature unquestionably demonstrate the recrystallization into its initial phase. However, at sufficiently high annealing temperatures (403 and 413 K), recrystallization to Form I, the thermodynamically stable phase, is observed. Please note that an onset of Form I recrystallization is already present at 393 K, and a broad feature is observed at the expected peak position of Form I (Figure 5c). Figure 6b gives the intensity of the (001) peak upon recrystallization into Form I, which has a considerably shorter induction time in comparison to the recrystallization into Form IV. Additionally, Form I shows an enhanced crystallization rate compared to Form IV thin films.

Typical recrystallization curves of all three solvates are depicted in Figure 6c. It is observed that Form IV shows the quickest reappearance whereas Forms V and VI show longer induction times and slower crystallization rates. Nevertheless, in all three cases, the initial phase reappears after the annealing procedure. Multiple measurements on the same sample gave comparable crystallization kinetics, which reveals that thermal history does not play any role in our observations.

**Figure 7:**
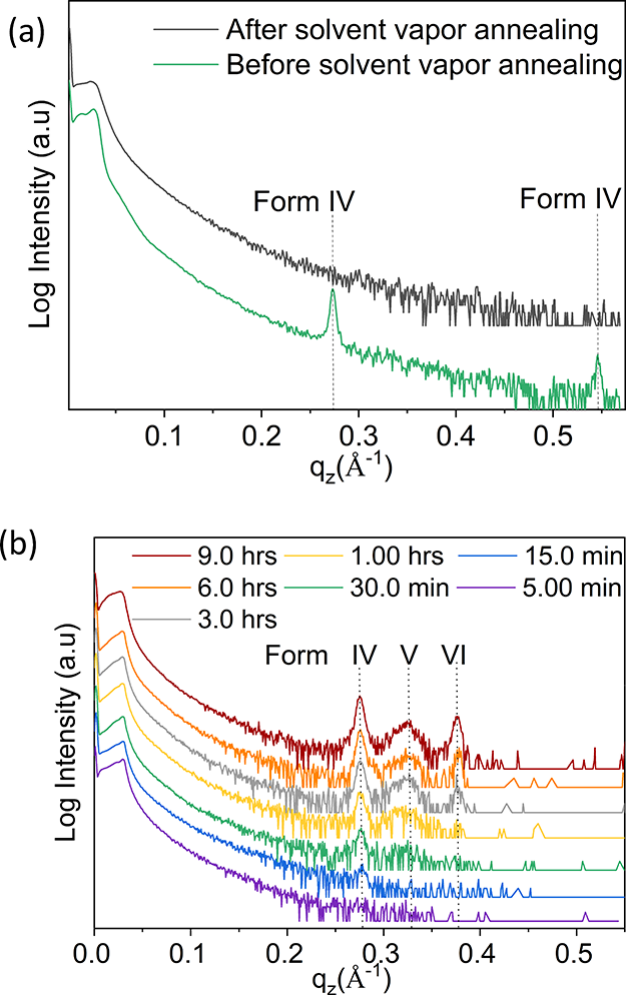
XRD patterns of the OEG-BTBT thin films. (a) Form IV film before and after solvent vapor annealing. (b) Crystallization of OEG-BTBT directly after drop casting at a temperature of 303 K reveals the successive appearance of the three solvates: Form IV, Form V, and Form VI as a function in time.

Solvent vapor annealing is a frequently used method to improve the crystalline quality of molecular thin films by inducing changes in the thin film morphology.^37,38^ In most cases, a transition from a metastable phase toward a stable phase is achieved.^39−41^ Furthermore, in amorphous films, access to specific polymorphs is potentially acquired by solvent vapor annealing.^42^ However, for our thin film systems, an unusual behavior is observed. As shown in Figure 7a, solvent vapor annealing of Form IV thin films with DCM results in the disappearance of the characteristic diffraction peaks. Moreover, solvent vapor annealing performed on Form I with DCM also resulted in the disappearance of the characteristic Bragg peaks (Figure S3). Furthermore, X-ray fluorescence measurements performed on the solvent vapor-annealed samples from DCM further detect the presence of chlorine atoms, thereby confirming the presence of the solvent molecules within the respective thin film sample (Figure S4). These results confirm the strong interactions of the solvent DCM with crystallized OEG-BTBT molecules, which suggests that diffusion of DCM into the crystalline system disrupts its molecular ordering. In contrast, solvent vapor annealing performed using chloroform as solvent barely modifies the morphology or crystallinity of Form I films (Figures S3 and S4).

As a conclusive step, thin films of OEG-BTBT were prepared by drop casting from DCM at 303 K, and within this film, we anticipated seeing all three forms (IV, V, and VI) coexisting together.^13^ Interestingly, the XRD investigations of the film in the as-prepared state (Figure 7b, purple curve) did not give any detectable Bragg peaks. The absence of diffraction peaks indicates that the film is in a rather disordered state after the deposition process. The same sample was uninterruptedly monitored for 24 h by continuous specular XRD scans to investigate the crystallization of the various solvates. The film slowly begins to solidify and crystallize under ambient conditions so that after 15 min, an onset of a diffraction peak was found first at 0.27 Å^–1^, which is assigned to Form IV. The peak intensity of the Form IV crystallites increases with time until an additional broad peak appears after 1 h at the position of Form V crystallites. After 3 h, the characteristic peak of Form VI emerges. Finally, a thin film composed of three coexisting phases is formed. The crystalline order thus appears at different induction times; we observe the quickest nucleation for Form IV, a slower nucleation process for Form V, and the slowest nucleation for Form VI. A similar trend is observed during in situ recrystallization experiments (Figure 6c). This is consistent with the fact that the phenomenon of memory effect is generally seen for systems that crystallize slowly (e.g., polymers), as is the case for our system of polymorphs.^21^

## Discussion

This work reports an outstanding memory effect from melt crystallization for the molecule OEG-BTBT. Three different solvates recrystallize in their initial crystal structure after melting the crystalline molecular packing and reaching the isotropic state at more elevated temperatures. Form IV has a melting temperature of 347 K and a clearing temperature of 376 K; nevertheless, the recrystallization into the same phase is observed after annealing up to a temperature of 393 K for 30 min. At an annealing temperature of 403 K, the system returns to its thermodynamic stable phase (Form I). Comparable observations were obtained with Form V and VI samples.

Unfortunately, details about the three crystal structures are not known, but the different fractions of DCM molecules within the crystal structure could be estimated.^13^ Based on the volume of the crystallographic unit cell and the required space of the involved molecules (OEG-BTBT and DCM), the number of DCM molecules per OEG-BTBT unit could be determined for each of the three solvates. In the case of Form IV, three DCM molecules are bound to a single OEG-BTBT molecule. Similarly, Form V and Form VI host two DCM molecules and one DCM molecule, respectively. The melting point and the isotropic transition temperature of these polymorphs follow the trend generally seen for solvates: the solvates have considerably lower melting points than the nonsolvated crystal structures, in full line with our system of polymorphs exhibiting a massive difference in melting points compared to the thermodynamically stable Form I (Table 1).

How to explain the origin of the melt memory effect in crystallization, i.e., that the initial phase reappears after annealing the sample above the isotropic transition temperature? The potentiality to recrystallize into the same initial form from melt can be correlated to the strong interactions of the OEG side chains with the solvent DCM. Irrespective of melting of the crystalline phase and losing any orientational order above the isotropic transition, the physical bonds between the OEG chains and DCM molecules remain. Cooling below the transition temperatures down to room temperature results in the regeneration of the initial phase since the structural elements—the OEG-BTBT molecules physically bonded with DCM molecules—are conserved in the molten state. These bonds are broken only by melting far above the isotropic transition temperature—in the case of Form IV at 403 K—which makes the nonsolvated crystal structure appear following the loss of the memory effects. Here, the significance of crystalline memory is that a specific temperature value above the respective melting point must be reached in order to get rid of the DCM fragments sticking onto the OEG chains. Only at sufficiently high temperatures do the bonds between the DCM molecules and the OEG side chain break, and the memory effect is lost.

How can the unusual behavior in solvent vapor annealing explain why the exposure to the solvent DCM impedes the appearance of a crystal structure? The reason might again originate from the strong interaction of the polar DCM molecules with the OEG side chains. It is well known that polyethylene glycol (PEG)—the corresponding polymer of OEG—is highly polar in nature and also tends to show high interactions with DCM due to its dipole moment.^43,44^ The Flory–Huggins interaction parameter between PEG and DCM is 1.67 at room temperature, indicating even better solubility at higher temperatures.^45^ Furthermore, molecular dynamics simulations reveal ample flexibility of the OEG side chains since the OC–CO group is preferred in the gauche arrangement.^19^ Hence, the wide variety of probable chain conformations, together with the strong polar character of the ethylene glycol chain, might be responsible for the potential ability of PEG to form host–guest systems like complex formation or crystalline inclusion compounds.^46−49^ Accordingly, the formation of multiple solvent phases for the OEG-BTBT molecule from the solvent DCM can be comprehended.

## Conclusions

This work demonstrates a melt memory effect for three solvates of the OEG-BTBT molecule with the solvent DCM. Phase-pure films of Forms IV, V, and VI were fabricated on silicon and glass substrates by solution processing. Subsequently, structural and morphological characterizations were performed to identify their unique characteristics. Hot-stage optical microscopy measurements revealed the respective isotropic transition temperatures of these solvates, which were well above the melting temperature of the crystalline state. Specular XRD measurements recorded after annealing the samples above the isotropic transition temperature showed recrystallization in the same initial phase, pointing to a crystalline melt memory effect. The reappearance of the same phase was observed after annealing well above the isotropization temperature. In the case of Form IV, the isotropic transition temperature is 376 K, but the films had to be annealed at 403 K to recrystallize into the thermodynamically stable phase (Form I). In-depth temperature-dependent in situ recrystallization experiments were conducted to measure induction times and crystallization kinetics. When going from Form IV to Form V and Form VI, an increasing induction time is observed together with a decreasing crystallization rate; by comparison, Form I shows an even shorter induction time and a quicker crystallization rate. The solvent vapor annealing procedure in DCM also provides unexpected results, since the structural ordering is disrupted and a transformation from a crystalline arrangement into a disordered one is observed. The strong interaction between the DCM molecules and the polar OEG chains of the molecule is most likely responsible for the observed effects; the penetration of the solvent into the crystalline state is expected to destroy the molecular packing. Moreover, these strong interactions keep the DCM molecules tightly bound to the OEG side chains even at elevated temperatures (above the isotropic temperature), which causes the observed crystalline melt memory effect. At sufficiently high temperatures, the DCM molecules are separated from the OEG chains so that crystallization in the thermodynamically stable phase takes place.

## Supporting Information Available

XRD patterns of films prepared on silicon wafers, XRD patterns of Form IV films after different annealing conditions, XRD before and after solvent vapor annealing, and X-ray fluorescence before and after solvent vapor annealing (PDF)
