# Supplementary material for: Memory Effect by Melt Crystallization Observed in Polymorphs of a Benzothieno-Benzothiophene Derivative
Source: Cryst Growth Des. 2023 Oct 16;23(11):8124–31. doi: 10.1021/acs.cgd.3c00847 (PMC10626567; doi:10.1021/acs.cgd.3c00847)
Supplement: Supplementary file 1 — cg3c00847_si_001.pdf [file cg3c00847_si_001.pdf]

Memory Effect by Melt Crystallisation Observed in Polymorphs of a Benzothieno-Benzothiophene Derivative.

Ann Maria James,[a] Alessandro Greco,[b] Félix Devaux, Nemo McIntosh,[c] Patrick Brocorens,[c] Jérôme Cornil,[c] Priya Pandey,[d] Birgit Kunert,[a] Lucia Maini,[d] Yves Henri Geerts,[e,f] Roland Resel,[a*]

[a] Institute of Solid State Physics, Graz University of Technology, Petersgasse 16, 8010 Graz, Austria
[b] Max Planck Institute for Polymer Research, 55128 Mainz, Germany
[c] Laboratory for Chemistry of Novel Materials, University of Mons, 7000 Mons, Belgium
[d] Dipartimento di Chimica "G. Ciamician", University Bologna, 40126 Bologna, Italy
[e] Laboratoire de Chimie des Polymères, Université Libre de Bruxelles (ULB), 1050 Bruxelles,
[f] International Solvay Institutes of Physics and Chemistry, Université Libre de Bruxelles, 1050 Bruxelles, Belgium
*corresponding author: roland.resel@tugraz.at

Additional complimentary experimental results acquired to support the findings and observations recorded in the main manuscript are listed below. The corresponding importance is mentioned in the main manuscript accordingly. A short description associated with each one of them is given below.

Figure S1 gives the specular X-ray diffraction pattern recorded on phase pure films of Form IV, V and VI deposited on the silicon substrate. Figure 2 of the main part of the paper presents the analogous measurements made on glass substrates. The potentiality to observe these polymorphs independently on silicon as well as glass substrate thereby accounts for their reproducibility.

Figure S2a) gives the specular X-ray diffraction patterns measured on phase pure Form IV films in the as-prepared, annealing and recrystallization stages. The as-prepared films were measured at 298 K. The annealing conditions were maintained for 30 minutes to achieve the equilibrium state, and subsequently measured the curve at 373 K. And the measurement recorded after cooling down the system to room temperature confirms the recrystallization into the same Form IV. Figure S2b gives the specular X-ray diffraction curves recorded on the as prepared as well as on the cooled sample after annealing above isotropic transition temperature at different temperature values. And Figure S2c gives the recrystallization behavior from 383 K at different annealing times.

Figure S3 illustrates the specular X-ray diffraction measurements made on phase pure Form I sample before and after solvent vapor annealing procedures with (a) dichloromethane and (b)chloroform, respectively. Analogous results on Form IV are demonstrated in Figure 6.

Figure S4 illustrates the X-ray fluorescence measurements made on phase pure Form I sample before and after solvent vapor annealing procedures with (a) dichloromethane and (b) chloroform, respectively.

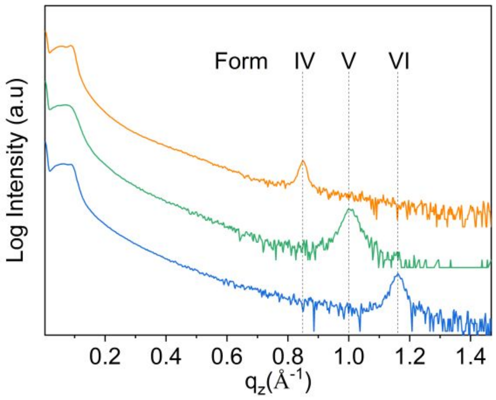

Figure S1: X-ray diffraction pattern of phase pure films of OEG-BTBT prepared on silicon wafers.

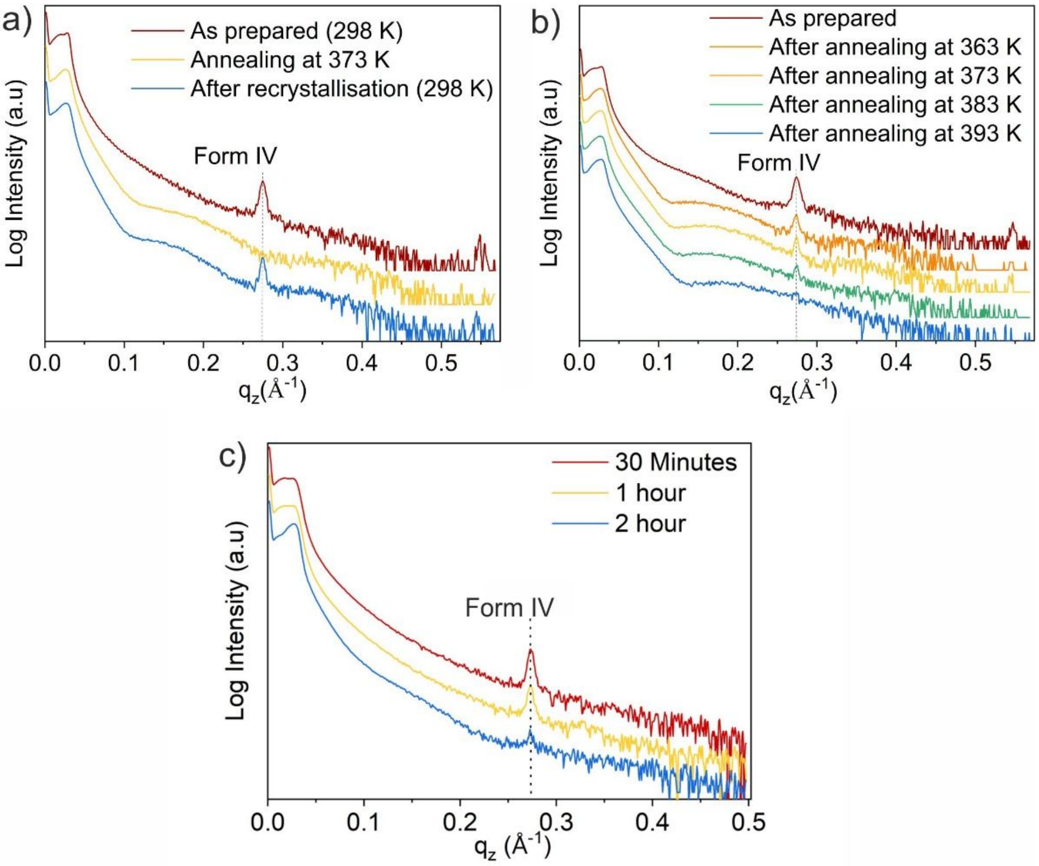

Figure S2: X-ray diffraction pattern of Form IV thin films a) before, during annealing at 373 K and after cooling down to room temperature and b) in the as prepared state and recrystallized after annealing at different temperatures and c) recrystallized from 383 K after different annealing times.

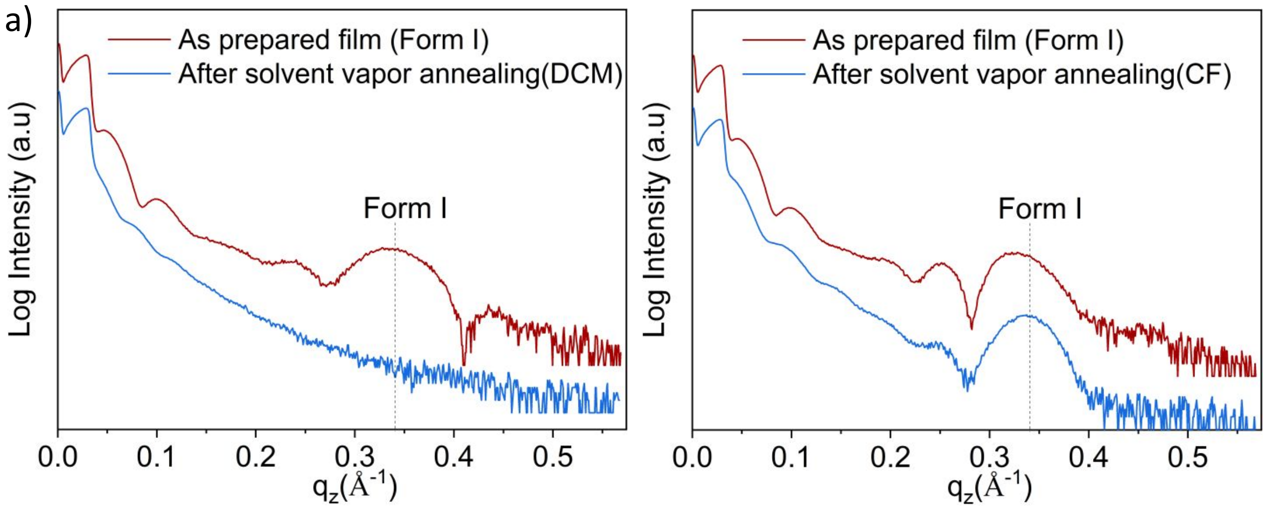

Figure S3: X-ray diffraction pattern recorded on solvent vapor annealed Form I samples using (a) dichloromethane and (b) chloroform.

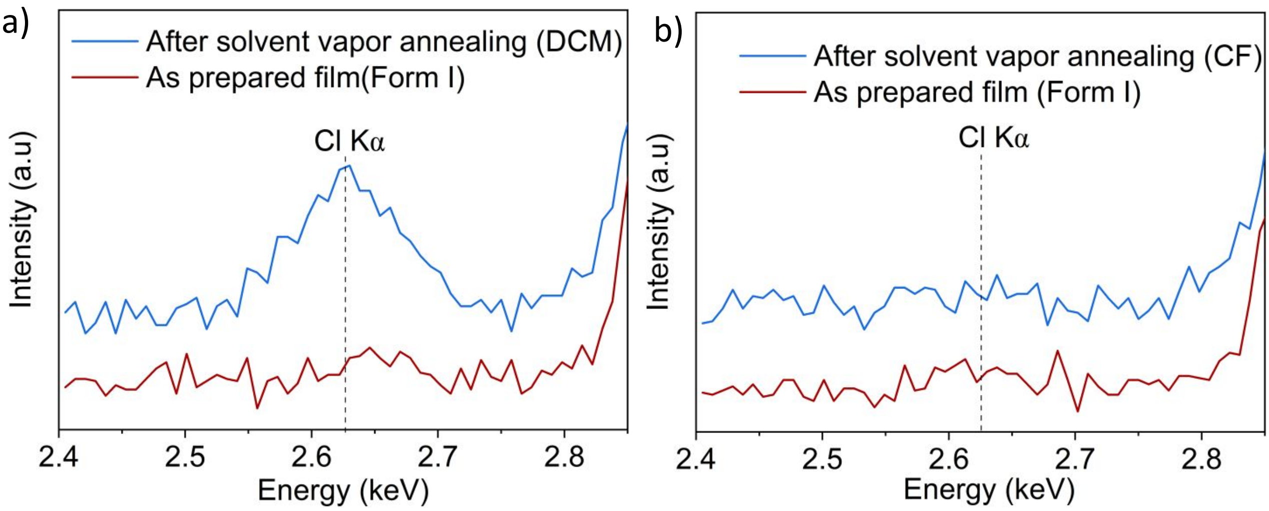

Figure S4: X-ray fluorescence spectra recorded on solvent vapor annealed Form I samples using (a) dichloromethane (b) chloroform.
